# The Diacylglycerol Kinase α/Atypical PKC/β1 Integrin Pathway in SDF-1α Mammary Carcinoma Invasiveness

**DOI:** 10.1371/journal.pone.0097144

**Published:** 2014-06-02

**Authors:** Elena Rainero, Cristina Cianflone, Paolo Ettore Porporato, Federica Chianale, Valeria Malacarne, Valentina Bettio, Elisa Ruffo, Michele Ferrara, Fabio Benecchia, Daniela Capello, Wolfgang Paster, Irene Locatelli, Alessandra Bertoni, Nicoletta Filigheddu, Fabiola Sinigaglia, Jim C. Norman, Gianluca Baldanzi, Andrea Graziani

**Affiliations:** 1 Integrin Biology Laboratory, Beatson Institute for Cancer Research, Glasgow, Scotland, United Kingdom; 2 Department of Translational Medicine, Università del Piemonte Orientale, Novara, Italy; 3 Sir William Dunn School of Pathology, University of Oxford, Oxford, United Kingdom; University of Bergen, Norway

## Abstract

Diacylglycerol kinase α (DGKα), by phosphorylating diacylglycerol into phosphatidic acid, provides a key signal driving cell migration and matrix invasion. We previously demonstrated that in epithelial cells activation of DGKα activity promotes cytoskeletal remodeling and matrix invasion by recruiting atypical PKC at ruffling sites and by promoting RCP-mediated recycling of α5β1 integrin to the tip of pseudopods. In here we investigate the signaling pathway by which DGKα mediates SDF-1α-induced matrix invasion of MDA-MB-231 invasive breast carcinoma cells. Indeed we showed that, following SDF-1α stimulation, DGKα is activated and localized at cell protrusion, thus promoting their elongation and mediating SDF-1α induced MMP-9 metalloproteinase secretion and matrix invasion. Phosphatidic acid generated by DGKα promotes localization at cell protrusions of atypical PKCs which play an essential role downstream of DGKα by promoting Rac-mediated protrusion elongation and localized recruitment of β1 integrin and MMP-9. We finally demonstrate that activation of DGKα, atypical PKCs signaling and β1 integrin are all essential for MDA-MB-231 invasiveness. These data indicates the existence of a SDF-1α induced DGKα - atypical PKC - β1 integrin signaling pathway, which is essential for matrix invasion of carcinoma cells.

## Introduction

Most cancer-associated mortality is caused by metastatic dissemination of primary tumors and the outgrowth of secondary tumors at distant sites. Among the microenvironment signals sustaining the invasive phenotype of cancer cells, stromal cell-derived factor-1α (SDF-1α, also named CXCL12), plays a major role in promoting cancer metastasis in several cancers, including breast cancer [1]. SDF-1α is a chemokine secreted by tumor-associated fibroblasts and bone marrow stromal cells, which through activation of its CXCR4 receptor, promotes migration and invasion of malignant cells and their homing to target organs [2], [3]. Indeed CXCR4 is a poor prognosis predictor in several cancer types [4].

In breast cancer, the chemotactic and invasive activity of SDF-1α/CXCR4 is mediated by both Gα_13_-mediated activation of RhoA and Gαi-mediated activation of Rac1 via DOCK180/ELMO, which regulate cytoskeletal remodeling [5], [6]. In myeloid cells, Rac1 mediates SDF-1α-induced increase of integrin affinity, while RhoA mediates formation of membrane protrusions and CXCR4 trafficking to the cell surface in Rab11+ endosomes [7], [8]. Moreover, in gastric cancer cells SDF-1α invasive and proliferative activity is also stimulated by Gαi- and PI3Kβ-mediated activation of mTOR complex 1, which contributes to Rac1 activation as well [9]. Finally, atypical protein kinases C (PKCζ and ι, hereafter aPKCs), which do not bind diacylglycerol (DG), play a key role in mediating chemotaxis of bone marrow and muscle stem cells, and of lymphocytes [10], [11]. However neither the mechanisms by which SDF-1α stimulates aPKCs nor their role in SDF-1α invasive signaling in breast cancer cells have been elucidated.

DGKs are a multigenic family of ten enzymes phosphorylating DG to generate phosphatidic acid (PA), thus reciprocally regulating in a highly compartmentalized manner the concentration of both lipid second messengers and their signaling activities [12]. Indeed, activation of DGKs results in the termination of DG-mediated signals, while triggering PA-mediated ones. Increasing evidence points to DGKα as a critical node in oncogenic signaling and as a putative novel therapeutic target in cancer: inhibition or silencing of DGKα has been shown to reduce tumor growth and mortality in glioblastoma and hepatic carcinoma xenograft models [13], [14]. Moreover, we recently showed that DGKα activity sustains the pro-invasive activity of metastatic p53 mutations, by promoting the recycling of α5β1 integrin to the tip of invasive protrusions in tridimensional matrix [15]. DGKα is activated and recruited to the membrane by growth factors, estrogen and tyrosine kinase oncogenes through Src-mediated phosphorylation. Upon growth factor stimulation, activation of DGKα mediates cell migration, invasion and anchorage-independent growth [16]–[21]. Indeed, activation of DGKα is a central element of a novel lipid signaling pathway involving PA-mediated recruitment at the plasma membrane and activation of aPKCs in a complex with RhoGDI and Rac1, thus providing a positional signal regulating Rac1 activation and association to the membrane [22], [23].

Altogether these data suggest that DGKα and aPKCs may act as signaling nodes in the molecular crosstalk between soluble chemotactic factors and the extracellular matrix, thus prompting us to investigate the involvement of DGKα in cell migration and invasion induced by SDF-1α in breast cancer cells. In here we show that upon SDF-1α stimulation of breast cancer cells, DGKα activity mediates aPKCs localization at protrusion sites and the subsequent recruitment of β1 integrin and MMP-9 secretion. Conversely over-expression of DGKα is sufficient to induce aPKCs-dependent cell elongation. Finally, we observed that the DGKα – aPKCs – β1 integrin pathway is an essential mediator of chemokine-promoted cell migration and matrix invasion.

## Materials and Methods

### Cells Culture and Reagents

MDA-MB-231 cells were from ATCC, 293FT were from Life Technologies. Cells were cultured in DMEM (Life Technologies) with 10% FCS (LONZA) and antibiotics/antimycotics (Sigma-Aldrich) in humidified atmosphere 5% CO_2_ at 37°C.

R59949 (Sigma-Aldrich) was dissolved in DMSO; equal amounts of DMSO were used in the control samples. All reagents are from Sigma-Aldrich apart: matrigel growth factor reduced (BD Bioscences), human recombinant SDF-1α and HGF (Peprotech), Myr-PKCζ/ι peptide inhibitor (BIOMOL) and NSC23766 (Tokris bioscience).

Antibodies: myc (clone 9E10 Santa Cruz), MMP-9 (2C3 Santa Cruz for western blotting and immunofluorescence or IC9111F RDsystems); PKCζ/ι (P0713 Sigma); β1 integrin (cat. 610467 BD Transduction Laboratories for western blotting and immunofluorescence or BV7 Abcam for cytofluorimetry); StrepMab-tag II (2-1507-001 IBA); actin (C-2 Santa Cruz); tubulin (DM1A Sigma-Aldrich); DGKα (Shaap et al., 1993), human RCP (rabbit in-house Ab raised against RCP residues 379–649); Cdc42 (2462 Cell signaling). Secondary antibodies HRP-mouse and HRP-rabbit were from Perkin Elmer. Secondary antibodies anti-rabbit Ig Alexa Flour-488 and anti-mouse Ig Alexa Flour-488 were from Life Technologies as well as Alexa Flour 546-phalloidin, TO-PRO-3 is from Life Technologies.

### Invasion Assay

Invasion assay were performed in BD BioCoat Matrigel Chambers. 50,000 cells/well were plated in the upper chamber whereas SDF-1α (100 ng/ml) or 10% FCS were added to the lower chamber in serum free medium. After 22 hours of incubation in a humidified atmosphere 5% CO_2_ at 37°C, non invading cells were removed from the upper surface of the membrane and invading cells were fixed and stained with Diff-Quik (Medion Diagnostic) before counting.

### Wound Healing Assay

Cells were grown to confluence in 12 wells plates and the monolayer wounded with a pipet tip. Cell debris were removed and monolayer maintained in serum free medium for 24 hours with or without HGF (50 ng/ml). The cells were stained with Diff-Quik (Medion Diagnostic) and for each experimental point 8 fields photographed (Axiovert inverted microscope with a 4x objective and a digital camera). Cells migrating inside 2.3 mm of wound were counted.

### DGKα Activation Assay

Cells homogenates were prepared by collecting the cells with a rubber scraper in buffer B (25 mM Hepes (pH 8), 10% glycerol, 150 mM NaCl, 5 mM EDTA, 2 mM EGTA, 1 mM ZnCl2, 50 mM ammonium molibdate, 10 mM NaF, 1 mM sodium orthovanadate and Protease Inhibitor Cocktail), homogenizing them with a 23 G syringe and by spinning at 500 g for 15 min. Protein concentration was determined by the bicinchoninic acid method (Pierce) and equalized for each point with buffer.

DGKα activity in cell homogenates (25 µl) was assayed by measuring initial velocities (5 min at 30°C) in presence of saturating substrates concentration (1 mg/ml diolein, 5 mM ATP, 3 µCi/ml γ^32^P-ATP (Perkin Elmer), 10 mM MgCl2, 1 mM ZnCl2, 1 mM EGTA in 25 mM Hepes pH 8, final reaction volume 50 ml). Reaction was terminated with 0.1 M HCl and lipids were extracted with cloroform methanol (1∶1). PA was separated by TLC in chloroform:methanol:water:25% ammonium hydroxide (60∶47∶11∶4). ^32^P-PA was identified by co-migration with PA standards stained by incubation in iodine chamber. Radioactive signals were detected and quantified by Molecular Imager (Bio-Rad).

### Immunofluorescence

Cells (30,000/well) were plated on matrigel coated coverlips in 24 wells cell culture plate and serum deprived for 16–24 hours before stimulation. After stimulation cells were washed with PBS, fixed in PBS containing 3% paraformaldehyde and 4% sucrose and permeabilized in cold Hepes-Triton buffer (20 mM Hepes, 300 mM sucrose, 50 mM NaCl, 3 mM MgCl2, 0.5% Triton X-100, pH 7.4). PBS containing 2% BSA was used as blocking reagent for 15 minutes and as diluting agent for primary and secondary antibodies (incubated for at least 1 hour). Intermediate washing was performed with PBS containing 0.2% BSA.

Antibodies were added directly onto each glass coverslip in a humidified chamber. Finally, each glass coverslip was washed briefly in water and mounted onto a glass microscope slide using Mowiol (20% Mowiol 4–88, 2.5% 1, 4-diazabicyclo [2.2.2] octane in PBS, pH 7.4).

Confocal images were acquired with Leica confocal microscope TCS SP2 using a 63x objective, NA = 1.32, equipped with LCS Leica confocal software. Basal planes are shown. Each experimental point was performed in duplicate. Depending on preparation quality in each replicate roughly 30 images were taken, containing between 70 and 100 cells.

### Morphometry

For cell length analysis cells were plated in 24 wells plates and phase contrast images of live cell were acquired with an Axiovert inverted microscope equipped with a 40x objective and a digital camera (Carl-Zeiss) and total cell length was measured with Image-Pro Plus software (MediaCybernetics). Alternatively in Fig. 6D and Fig. S5B we used a 10x Plan Fluor objective, NA 0.3, and an inverted microscope (TE200; Nikon) with a digital camera (CoolSNAP HQ; Photometrics) and Metamorph software (Molecular Devices). For each experimental condition 5 random fields were photographed containing more than 100 cells.

### Cytofluorimetry

Cells were detached with ice could PBS 4 mM EDTA, fixed with PBS containing 3% paraformaldehyde and stained as indicated for 30 min. After washing with PBS containing 0.2% BSA cells were analyzed with a FACScalibur instrument an CellQuest software (BD) or Flowing software (Turku Bioimaging).

### siRNA for Transient Silencing

Transient silencing was obtained by transfection of siRNA (Sigma Genosys or Life Technologies). Briefly were plated on matrigel coated coverlips to 30–50% confluence the day before transfection and transfected using lipofectamine 2000 (Life Technologies) according to manufacturer’s instructions. The day after transfection cells were serum deprived for further 18 hours before immunofluorescences or western blotting.

Validated siRNA DGKα [17] sense 5′ GGAUGGCGAGAUGGCUAAAtt 3′ antisense 5′UUUAGCCAUCUCGCCAUCCgg 3′.

siRNA PKCζ sense 5′CGUUCGACAUCAUCACCGAtt3′antisense 5′UCGGUGAUGAUGUCGAACGgg3′.

siRNA PKCι sense 5′CGUUCGACAUCAUCACCGAtt3′ antisense 5′UCGGUGAUGAUGUCGAACGgg3′.

siRNA β1 integrin sense 5′GGAGGAAUGUUACACGGCU3′ antisense 5′ AGCCGUGUAACAUUCCUCCag 3′.

siRNA RCP: ON-TARGETplus RAB11FIP1 siRNA L-015968-00-0005 (Dharmacon). Silencer negative control siRNA AM4611 (Life Technologies) was used as negative control.

### Generation of Tet-inducible Strep-tagged DGKα Construct and Cell Infection

Human DGKα was amplified from pMT2- DGKα [24] by PCR using the primers DGKα_ScII_fw (5′-CCGCGGGCAGCATGGCCAAGGAGAGGGGC-3′) and DGKα_H3_rv (5′-AAGCTTTTAGCTCAAGAAGCCAAA-3′) and cloned into pEXPR-IBA-105 (IBA GmbH) via SacII and HindIII to generate pEXPR-Strep-DGKα. In a further step Strep-DGKα was amplified by PCR using primers IBA_fw_N1 (5′-GCGGCCGCAGACCCACCATGGCTAGC-3′) and 105DGKa_MluI_rv (5′-ACGCGTTTAGCTCAAGAAGCCAAA-3′) and cloned via NotI and MluI to pLVX-Tight-Puro (Clontech). All constructs were verified by DNA sequencing.

The resulting pLVX-Tight-PURO-OST-DGKα presents OST- DGKα after a tetracycline controlled promoter and was used with the Lenti-X Tet-On Advanced Inducible Expression System (Clontec) according to manufacturer’s instruction. Lentiviral particles were obtained in 293FT packaging cells co-transfected with helper vectors. After double infection and selection we obtained a polyclonal population of MDA-MB-231 cells expressing OST-DGKα in a tetracycline inducible manner. A control cell line was also generated with an empty vector.

### Generation of MDA-MB-231 Stably Expressing Myc-DGKa

Myc-DGKα was amplified from PMT2-myc-DGKα [16] by PCR using the primers sense.

5′CTCGAGACCAATGGAACAAAAGTTGATTTCAGAAGAAGATTTATTAATGGCCAAGGAGG3′, antisense 5′GCCCCTCTCCTTGGCCATTAATAAATCTTCTTCTGAAACAACTTTTGTTCCATGGCTCGAGTGCA3′ and cloned in the pDONOR211 vector using the Gateway system (Life Technologies) according to manufacturer’s instructions. The Gateway Technology (Life Technologies) was also used to subclone myc-DGKα into pLenti4/V5-DEST lentiviral vector. Lentiviral particles were obtained in 293FT packaging cells co-transfected with helper vectors. After infection and selection we obtained a polyclonal population of MDA-MB-231 cells constitutively expressing myc-DGKα.

### Inducible Silencing of DGKα in MDA-MB-231

We used the commercial pTRIPZ Inducible Lentiviral Human DGKA shRNA Clone ID: V3THS_340705 (shRNA-DGKα1) or pTRIPZ Inducible Lentiviral Non-silencing shRNA Control RHS4743 (shRNA-CTRL). Those vectors express shRNA and turboRFP under a doxycycline regulated promoter (Thermo Scientific Open Biosystems). Lentiviral particles were obtained in 293FT packaging cells co-transfected with helper vectors. After infection and selection we obtained a polyclonal population of MDA-MB-231 cells which upon induction with doxycycline (1 µg/ml, 72 hours) are 100% RFP positive.

### Stable Silencing DGKα in MDA-MB-231

The shRNA for DGKα (forward: 5′ GATCCCCGGTCAGTGATGTCCTAAAGTTCAAGAGACTTTAGGACATCACTGACCTTTTTGGAAA reverse: 5′ AGCTTTTCCAAAAAGGTCAGTGATGTCCTAAAGTCTCTTGAACTTTAGGACATCACTGACCGGG) was cloned with H1-Promoter within the lentiviral vector pCCL.sin.PPT.hPGK.GFPWpre [25]. The resulting vector co-express shRNA-DGKα and GFP (shRNA-DGKα2). Empty vector was used as a control. Lentiviral particles were obtained in 293FT packaging cells co-transfected with helper vectors (Life Technologies). At 1 week after infection nearly 100% of cells were GFP^+^.

### Generation of ShRNA- β1 Integrin MDA-MB-231

ShRNA-β1integrin in pLKO were a kind gift of P. Defilippi [26]. Lentiviral particles were generated with Sigma Mission Lentivaral packaging mix according to manufacturer’s instruction in 293FT cells and selected with puromycin. Empty pLKO was used as a control.

### Western Blotting

To verified protein down-regulation cells were lysed 48 hours after transfection. Cell were washed with ice cold PBS, scraped on ice in lysis buffer (25 mM Hepes, pH 8, 150 mMNaCl, 0.5/1% Nonidet P-40, 5 mM EDTA, 2 mM EGTA, 1 mM ZnCl2, 50 mM NaF, 10% glycerol supplemented with fresh 1 mM Na_3_VO_4_, and protease inhibitors) and clarified after centrifugation of 15 minutes at 12000 rpm at 4°C. Samples were then resuspended in Laemmli buffer, heat denatured, and separated by SDS/PAGE. Proteins were then transferred on PVDF membrane by using semi-dry system. Membrane was then blocked with 5% BSA in PBS and incubated at 4°C overnight with primary antibodies diluted in TBS tween 0.1%, BSA 2%, 0.01% azide. After 4 washes with TBS-Tween 0.1%, membranes were incubated with secondary antibodies and washed again. Western blot were visualized using Western Lightning Chemiluminescence Reagent Plus (Perkin Elmer).

### Quantitative RT-PCR

RNA was extracted by TRI-Reagent Solution (Life Technologies) retrotrascribed with High-Capacity cDNA Reverse Transcription Kits (Life Technologies) and cDNA quantified by real time PCR using GUSB as normalizer. TaqMan gene expression assays we from Life Technologies: β1 integrin (Hs 00559595), GUSB (Hs 00939627), DGKα (Hs 00176278) and MMP-9 (Hs 00234579).

### MMP-9 Secretion

MDA-MB-231 cells (250,000 cells/well) were plated in 6-well cell culture plate and transfected with the indicated siRNA. After 24 hours in serum free media cells were treated with SDF-1α (100 ng/ml in 500 µl serum-free medium). After 24 hours the MMP-9 concentration in the supernatants was determined by ELISA assay (Life Technologies).

### Statistical Analysis

Data are shown as the mean ± SEM. For statistical analysis, Student’s t-test or *ANOVA* were used. Experiments shown are representative at least 3 independent experiments.

## Results

### DGKα Is Necessary for SDF-1α-induced Cell Invasion

**Figure 1:**
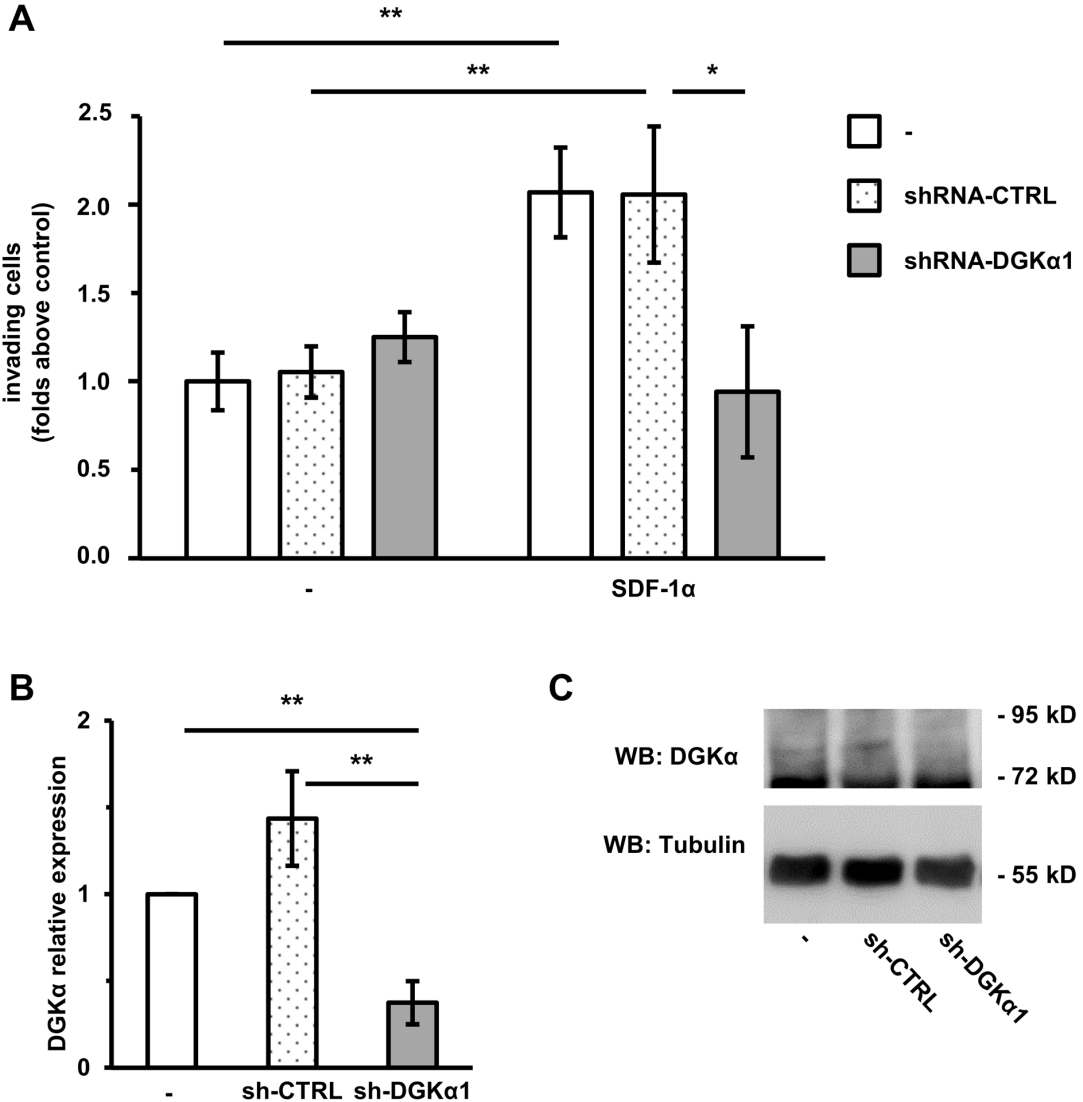
DGKα is necessary for SDF-1α-induced cell invasion. MDA-MB-231 cells were infected with lentiviral vectors expressing an inducible shRNA against DGKα (shRNA-DGKα1) or an inducible control shRNA (shRNA-CTRL). Parental and infected cells were treated with 1 µg/ml doxycycline for 72 hours to promote shRNA transcription. A) 50,000 cells were plated on matrigel invasion chamber and incubated for 24 hours in presence or in absence of SDF-1α (100 ng/ml). Histogram reports mean ± SE of fold over control values from 3 independent experiments with *t-test p<0.05, **t-test p<0.01. B) The efficiency of DGKα down–regulation by shRNA was verified by quantitative RT-PCR. **t-test p<0.01. A) Cells were lysed and the efficiency of DGKα down–regulation by shRNA was verified by western blot, tubulin was used as a loading control.

We previously showed that DGKα is necessary for matrix invasion promoted by Epidermal Growth Factor (EGF) [15] or Hepatocyte Growth Factor (HGF) in MDA-MB-231 breast carcinoma cells [27]. In order to investigate the role of DGKα in chemokine invasive signaling in breast cancer, we knocked down DGKα in MDA-MB-231 using a lentiviral construct expressing a DGKα-specific shRNA under an inducible promoter (shRNA-DGKα1). This construct strongly downregulated DGKα expression when compared with parental cells or a non-targeting control sequence (shRNA-CTRL, Fig. 1 B and C). The invasive ability of parental, DGKα-knocked down and control cells were evaluated in a Matrigel invasion assay. SDF-1α (100 ng/ml) doubles the number of parental as well as shRNA-CTRL MDA-MB-231 invading across the matrigel insert (Fig. 1 A). Conversely, shRNA-DGKα1 cells were unresponsive to SDF-1α stimulation. We confirmed this finding with an independent shRNA (shRNA-DGKα2) giving a comparable inhibition of SDF-1α stimulated matrix invasion (Fig. S1), making off-target effects unlikely.

Those findings indicates that DGKα mediates the pro-invasive signaling promoted not only by tyrosine kinase receptors [22] but also by chemokine receptors involved in tumor cells metastatization, such as those of SDF-1α.

### SDF-1α Stimulates DGKα Activity and Localization at Protrusions Sites

The previous findings that HGF, EGF and VEGF activate DGKα and promote its recruitment to the plasma membrane in epithelial and endothelial cells [15], [17], [22] suggest that SDF-1α may promote localized DGKα activation at ruffling sites. Despite its biological significance, the low level of DGKα expression in MDA-MB-231 cells hampers activation and localization studies of the endogenous protein with currently available antibodies.

**Figure 2:**
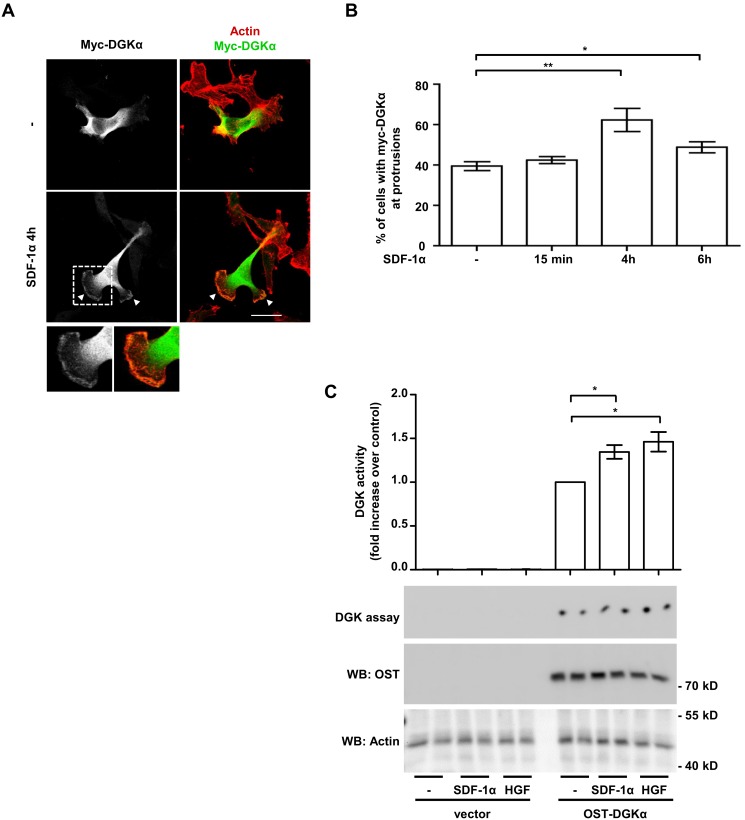
SDF-1α stimulates DGKα activity and localization at protrusions site. A) MDA-MB-231 cells, stably expressing myc-DGKα, were plated on matrigel-coated coverslips for 20 hours in FCS containing medium and cultured for further 20 hours in serum free medium. Cells were then stimulated with 50 ng/ml of SDF-1α for the indicated times, fixed and stained for actin (red) and myc-DGKα (green). Representative images at 4 hours after stimulation. Arrowheads indicate DGKα at protrusions. Histogram (B) reports the percentage of cells displaying myc-DGKα at protrusion as mean ± SE of 5 independent experiments, *t-test p<0.05, **t-test p<0.005. Scale bar 24 µm. C) MDA-MB-231 cells were infected with a lentiviral vector expressing inducible OST-tagged DGKα or an empty vector. To induce DGKα expression, cells were treated overnight with doxycycline (1 µg/ml) in serum free medium. Cell were homogenized with buffer B in absence of detergent and analysed for DGK activity (upper panel). Values are mean ± SE of 4 independent experiments with *t-test p<0.05. OST-DGKα and actin protein expression was verified by anti-OST and anti-actin western blot (lower panel).

Thus, for localization studies, MDA-MB-231 cells were stably infected with a lentiviral vector expressing myc-DGKα and plated on matrigel-coated coverslip to mimic the epithelial microenvironment. In unstimulated serum-deprived cells, myc–DGKα was mainly cytoplasmic, with some cells displaying very little accumulation at cell protrusions (Fig. 2A). Prolonged SDF-1α stimulation (50 ng/ml; 4 to 6 hours) resulted in the localization of DGKα at the tip of large protrusions (Fig. 2A and B). No detectable changes were observed at earlier time points (15 minutes, Fig. 2B).

For enzymatic activation assays, we infected MDA-MB-231 with a lentiviral vector expressing OneStrep-Tagged DGKα (OST-DGKα) under the control of a doxycycline-inducible promoter. Upon 48 hours doxycycline treatment (1 µg/ml), OST-DGKα was strongly overexpressed as compared to endogenous protein (Fig. S2A). Under these conditions the enzymatic activity of OST-DGKα was responsible for almost the entire DGK activity measured in cell homogenates. Both SDF-1α and HGF (a well known DGKα activator) induced a further moderate increase of OST-DGKα activity within 15 minutes of stimulation (Fig. 2C).

Altogether these data indicate that SDF-1α regulates DGKα activity and localization and suggest that DGKα plays a role in the formation and/or extension of cell protrusions induced by SDF-1α.

### DGKα Mediates SDF-1α-induced Cell Invasion by Regulating aPKCs Recruitment to Cell Protrusions

**Figure 3:**
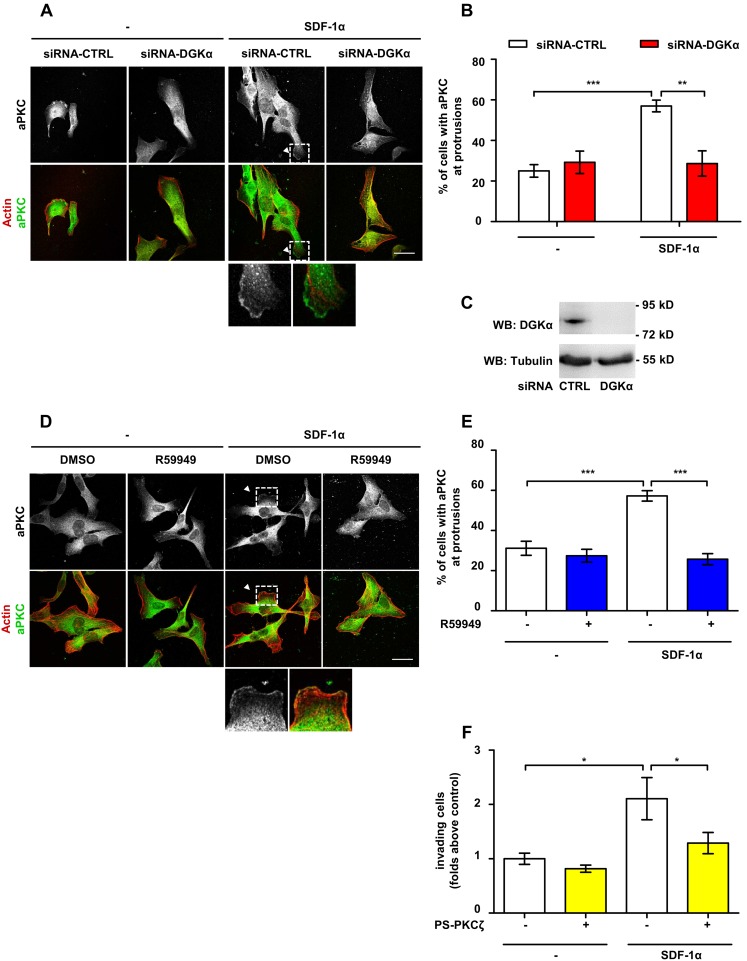
DGKα mediates SDF-1α-induced cell invasion by regulating aPKCs recruitment to cell pseudopods. A) MDA-MB-231 cells were plated on matrigel-coated coverslips for 20 hours in FCS containing medium, transfected with CTRL or DGKα –specific siRNA and cultured for further 20 hours in serum free medium. Cells were then stimulated for 6 hours with 50 ng/ml SDF-1α, fixed, and stained for actin (red) and aPKCs (green). Arrowhead indicates aPKCs at protrusions. Scale bar 24 µm. B) Histogram reports the percentage of cells displaying aPKCs at protrusions as mean ± SE of 3 independent experiments with **t-test p<0.005, ***t-test p<0.0005. C) MDA-MB-231 cells were transfected with CTRL or DGKα –specific siRNA and lysed. The efficiency of DGKα down–regulation by siRNA was verified at 48 hours after transfection by western blot, tubulin was used as loading control. D) MDA-MB-231 cells were plated on matrigel-coated coverslips for 20 hours in FCS containing medium and cultured for further 20 hours in serum free medium. Cells were then stimulated for 6 hours with 50 ng/ml SDF-1α, in presence or in absence of 1 µM R59949, fixed and stained for actin (red) and aPKCs (green). Arrowheads indicate aPKCs at protrusions. Scale bar 24 µm. E) Histogram reports the percentage of cells displaying aPKCs at protrusions as mean ± SE of 3 independent experiments with ***t-test p<0.0005. F) MDA-MB-231 cells (10^6^/well) were plated on matrigel invasion chamber and stimulates for 24 hours with SDF-1α (50 ng/ml) in presence or absence of PKCζ pseudosubstrate (PS-PKCζ, 10 µM). Histogram reports mean ± SE of folds over control values from 3 independent experiments with *t-test p<0.05.

DGKα, by producing PA, mediates aPKCs activation and recruitment to the cell surface induced by growth factors [23], [28]. Thus, we set to investigate whether DGKα mediates SDF-1α-induced cell invasion by regulating aPKCs. To investigate the role of DGKα in regulating aPKCs localization, MDA-MB-231 cells were transiently transfected with control (siRNA-CTRL) or DGKα-specific siRNA (siRNA-DGKα). Upon 48 hours from transfection with siRNA-DGKα, the expression of DGKα was nearly undetectable as compared to its expression in cells transfected with control siRNA (Fig. 3C). Then, MDA-MB-231 cells were plated on matrigel-coated coverslips, serum starved and stimulated with 50 ng/ml SDF-1α for 6 hours. In control siRNA transfected cells, SDF-1α treatment significantly increased the percentage of cells displaying aPKCs at protrusions, while DGKα silencing strongly impaired aPKCs recruitment to the membrane (Fig. 3A and B). In order to verify the requirement for DGKα enzymatic activity, we carried out aPKCs localization assays in presence or in absence of 1 µM R59949, a rather specific DGKα inhibitor [16], [29]. R59949 treatment completely abrogated aPKCs localization at protrusions induced by SDF-1α, while it did not affect aPKCs localization in unstimulated cells (Fig. 3D and E).

In order to investigate the role of aPKCs in SDF-1α-induced invasion through extracellular matrix, MDA-MB-231 cells were treated with 10 µM cell permeable PKCζ pseudosubstrate (PS-PKCζ). In a matrigel invasion assay aPKCs inhibition significantly reduced SDF-1α-induced invasion, while basal invasion was unaffected in unstimulated cells (Fig. 3F).

Altogether, these data demonstrate that in SDF-1α-stimulated breast carcinoma cells, localized activity of DGKα at pseudopodial tips provides a crucial localization lipid signal for aPKCs recruitment, thus mediating SDF-1α-induced invasive signaling.

### DGKα and aPKCs Mediate SDF-1α-induced Recruitment of β1 Integrin to Protrusions Sites

**Figure 4:**
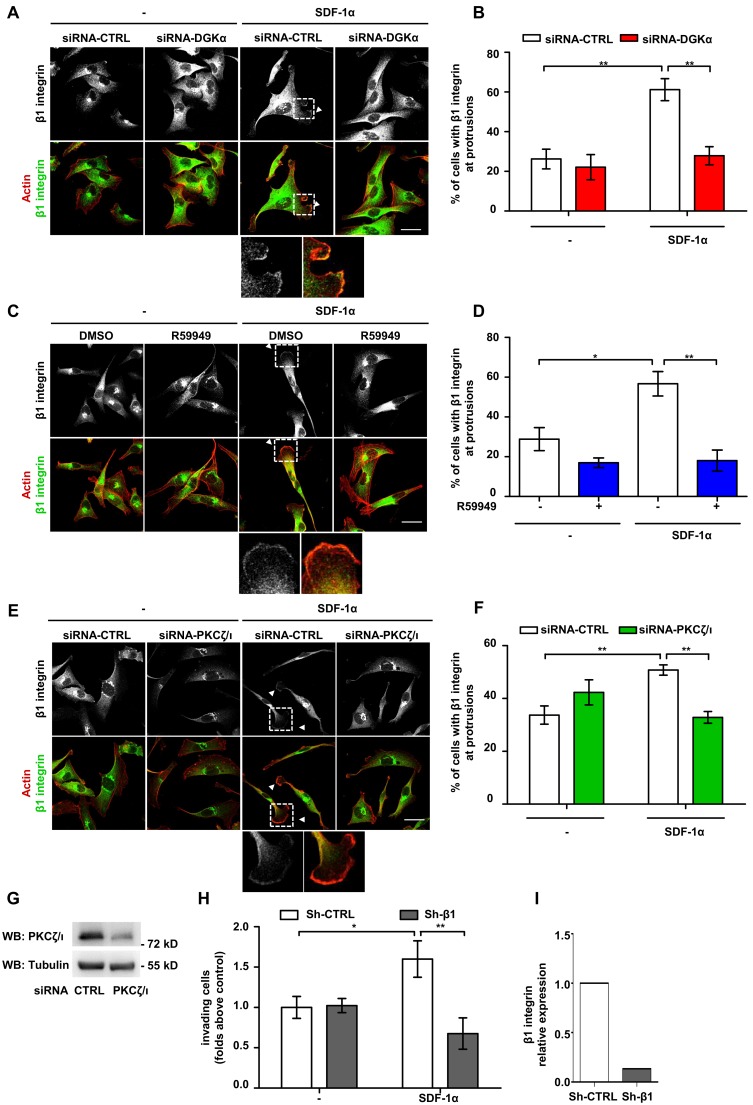
DGKα and aPKCs mediate SDF-1α-induced recruitment of β1 integrin to pseudopods. A) MDA-MB-231 cells were plated on matrigel-coated coverslips for 20 hours in FCS containing medium, transfected with CTRL or DGKα–specific siRNA and cultured for further 20 hours in serum free medium. Cells were then stimulated for 6 hours with 50 ng/ml SDF-1α, fixed and stained for actin (red) and β1 integrin (green). Arrows indicate β1 integrin at protrusions. Scale bar 24 µm. B) Histogram reports the percentage of cells displaying β1 integrin at protrusions as mean ± SE values of 3 independent experiments with **t-test p<0.005. C) MDA-MB-231 cells were plated on matrigel-coated coverslips for 20 hours in FCS containing medium and cultured for further 20 hours in serum free medium. Cells were then stimulated for 6 hours with 50 ng/ml SDF-1α, in presence or in absence of 1 µM R59949, fixed and stained for actin (red) and β1 integrin (green). Arrow indicates β1 integrin at protrusions. Scale bar 24 µm. D) Histogram reports the percentage of cells displaying β1 integrin at protrusions as mean ± SE of 3 independent experiments with *t-test p<0.05, **t-test p<0.005. E) MDA-MB-231 cells were plated on matrigel-coated coverslips for 20 hours in FCS containing medium, transfected with CTRL or PKCζ/ι –specific siRNA and cultured for further 20 hours in serum free medium. Cells were then stimulated for 6 hours with 50 ng/ml SDF-1α, fixed and stained for actin (red) and β1 integrin (green). Arrowheads indicate β1 integrin at protrusions. Scale bar 24 µm. F) Histogram reports the percentage of cells displaying β1 integrin at protrusions as mean ± SE of 3 independent experiments with **t-test p<0.005. G) MDA-MB-231 cells were transfected with CTRL and PKCζ/ι –specific siRNA and lysed. The efficiency of PKCζ/ι down–regulation by siRNA was verified by western bloting, tubulin was used as a loading control. H) MDA-MB-231 cells were infected with lentiviral vectors expressing a shRNA against β1-integrin (shRNA-β1) or a control sequence (shRNA-CTRL). 50,000 cells were plated on matrigel invasion chamber and incubated for 24 hours in presence or in absence of SDF-1α (100 ng/ml). Histogram reports mean ± SE of fold over control values from 3 independent experiments with *t-test p<0.05, **t-test p<0.01. I) The efficiency of β1-integrin down–regulation by shRNA was verified by quantitative RT-PCR.

Recycling and clustering of β1 integrin at the tip of invasive pseudopods is a key event sustaining the invasive properties of malignant cells [30]. Conversely, growth factors stimulate invasion both by inducing integrin clustering at actin-rich adhesive sites and lamellipodia and by stimulating integrin recycling [26], [31]. Thus, we set to investigate whether the DGKα and aPKCs at protrusions promote local accumulation of β1 integrin. In serum starved MDA-MB-231 cells plated on matrigel-coated coverslips β1 integrin is mostly localized in intracellular vesicles in the perinuclear/Golgi area. Upon SDF-1α stimulation, β1 integrin also localized in clusters at the tip of cell protrusions (Fig. 4A, C and E). However, either siRNA-mediated silencing of DGKα or R59949-mediated inhibition of its enzymatic activity impaired SDF-1α-induced localization of β1 integrin at cell extensions (Fig. 4A, B, C and D). Interestingly SDF-1α stimulation and DGKα inhibition did not affect the expression of β1 integrin at the cell surface, as measured by FACS analysis (Fig. S4A). Since DGKα promotes Rac1 activation and membrane ruffles by regulating aPKCs [15] and as DGKα mediates SDF-1α-induced aPKCs recruitment to the membrane protrusions, we assessed whether aPKCs controls β1 integrin localization. Indeed, siRNA-mediated silencing of aPKCs (Fig. 4G) impaired SDF1-α-induced localization of β1 integrin at cell protrusions (Fig. 4E and F).

Altogether these data suggest that SDF-1α, by activating the DGKα/aPKCs pathway, stimulates the clustering of β1 integrin at cell protrusions, rather than stimulating its bulk translocation at the plasma membrane.

Since the expression of constitutively-membrane bound myr-DGKα stimulates cell invasion by triggering RCP-mediated recycling of integrin α5β1 [15], we set to investigate the role of β1 integrin in SDF-1α-promoted cell invasion. To this purpose we used shRNA mediated knockdown of β1 integrin which resulted in an 80% reduction of its expression in MDA-MB-231 cells (Fig. 4I). We found that, β1 integrin knock down severely impaired the ability of MDA-MB-231 cells to invade through matrigel in response to SDF-1α stimulation (Fig. 4H).

Altogether these data indicate that DGKα, by regulating aPKCs, controls chemokine-induced β1 integrin localization at protrusion sites in breast carcinoma cells, thus confirming the pivotal role of β1 integrin in SDF-1α-promoted matrix invasion.

### DGKα and aPKCs Mediate SDF-1α-induced MMP-9 Secretion and Localization at Protrusions

Secretion of matrix metalloproteinases (MMPs) is involved in the extracellular matrix degradation required for invasion of cancer cells [32], [33]. SDF-1α stimulates the secretion of MMP-9 in several cancer cells, including MDA-MB-231 cells [34], [35]. In migrating cells, MMP-9 is addressed to the cellular extensions involved in cell migration and accumulates at their tips [36]. Thus, we investigated whether SDF1-α regulates intracellular localization and secretion of MMP-9 through the DGKα/aPKCs axis.

**Figure 5:**
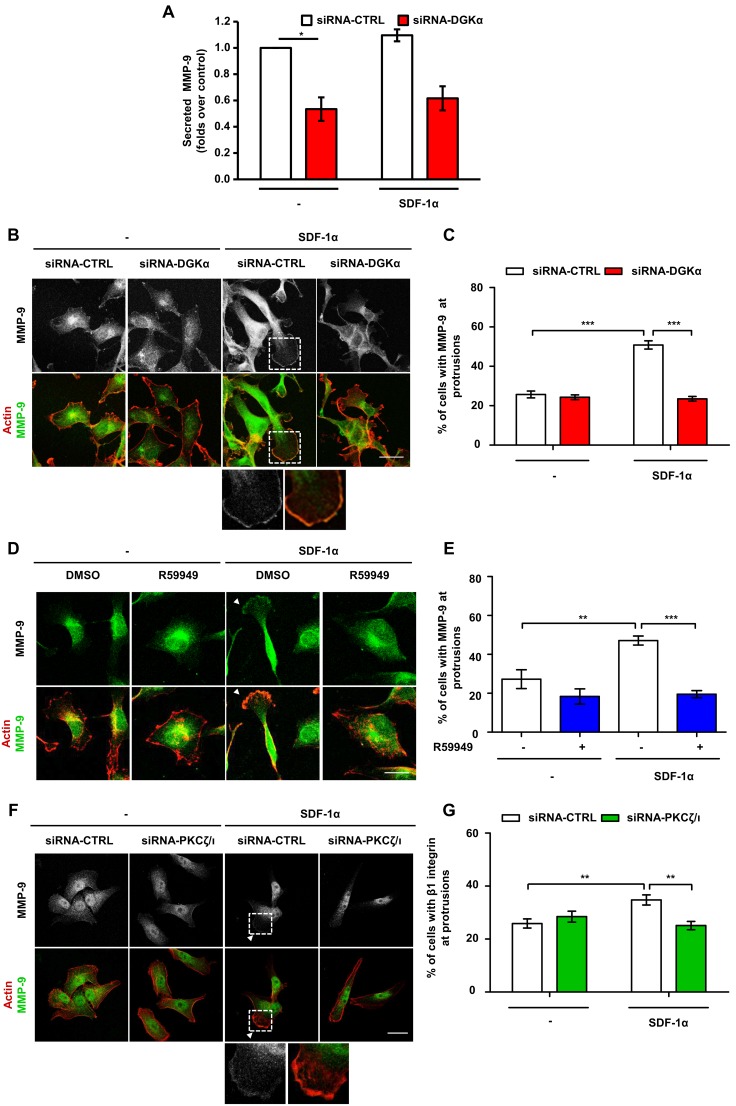
DGKα and aPKCs mediates MMP-9 secretion and localization at protrusions. A) MDA-MB-231 cells were transfected with CTRL or DGKα –specific siRNA and shifted to serum free media. After 24 hours cells were treated with 100 ng/ml SDF-1α in serum free medium for further 20 hours. MMP9 content in the supernatants was measured by ELISA assay, histogram reports secreted MMP-9 as mean ± SE of 3 independent experiments normalized for control, with *t-test p<0.05. B) MDA-MB-231 cells were plated on matrigel-coated coverslips for 20 hours in FCS containing medium, transfected with CTRL or DGKα –specific siRNA and cultured for further 20 hours in serum free medium. Cells were stimulated for 6 hours with 50 ng/ml SDF-1α, fixed and stained for actin (red) and MMP-9 (green). Arrowhead indicates MMP-9 at protrusions. Scale bar 24 µm. C) Histogram reports the percentage of cells displaying MMP-9 at protrusions as mean ± SE of 3 independent experiments with ***t-test p<0.0005. D) MDA-MB-231 cells were plated on matrigel-coated coverslips for 20 hours in FCS containing medium and cultured for further 20 hours serum free medium. Cells were stimulated for 6 hours with 50 ng/ml SDF-1α, in presence or in absence of 1 µM R59949, fixed and stained for actin (red) and MMP-9 (green). Arrowhead indicates MMP-9 at protrusions. Scale bar 24 µm. E) Histogram reports the percentage of cells displaying MMP-9 at protrusions as mean ± SE of 3 independent experiments with **t-test p<0.005, ***t-test p<0.01. F) MDA-MB-231 cells were plated on matrigel-coated coverslips for 20 hours in FCS containing medium, transfected with CTRL or PKCζ/ι –specific siRNA and cultured for further 20 hours in serum free medium. Cells were then stimulated for 6 hours with 50 ng/ml SDF-1α, fixed and stained for actin (red) and MMP-9 (green). Arrowhead indicates MMP-9 at protrusions. Scale bar 24 µm. G) Histogram reports the percentage of cells displaying MMP-9 at protrusions as mean ± SE of 3 independent experiments with *t-test p<0.05, **t-test p<0.005.

MDA-MB-231 cells presented a low, constitutive secretion of MMP-9 (40–80 pg/ml in the supernatant), which was not affected by SDF-1α but was severely reduced by siRNA-mediated silencing of DGKα (Fig. 5A). However, the mRNA levels of MMP-9 were not affected by either SDF-1α stimulation or DGKα inhibition, suggesting that this pathway does not regulate MMP-9 at the transcriptional level in these cells (Fig. S4C). Conversely, SDF-1α stimulated MMP-9 accumulation at protrusions of serum-starved MDA-MB-231 plated on matrigel-coated coverslips (Fig. 5B to E). We cannot rule out that MMP-9 staining may be associated to the plasma membrane, indeed FACS analysis of these cells detected low amounts of membrane-bound MMP-9 with a small increase in MMP-9 surface positive cells following SDF-1α stimulation (Fig. S4B). Silencing of DGKα impaired MMP-9 translocation induced by SDF-1α, while it did not affect its localization in unstimulated cells (Fig. 5B and C). Similarly, DGKα pharmacological inhibition with R59949, completely impaired MMP-9 recruitment induced by SDF-1α (Fig. 5D and E).

Altogether these data suggest that DGKα is essential for MMP-9 accumulation at protrusions and subsequent release in the extracellular space. Given the role of DGKα in regulating aPKCs, we investigated whether aPKCs mediates SDF-1α-induced regulation of MMP-9 localization. Indeed, siRNA-mediated silencing of aPKCs blunted SDF-1α induced MMP-9 localization at pseudopodial tips (Fig. 5F and G).

Altogether these data demonstrate that activation of the DGKα/aPKCs pathway drives both MMP-9 and β1 integrin localization at the pseudopodial tips, thus regulating the extension of invasive protrusions and sustaining the invasive behavior of MDA-MB-231 cells.

### DGKα Overexpression Promotes aPKC/Rac Dependent Cell Elongation

We observed that prolonged SDF1α treatment (6 hours, 50 ng/ml) of matrigel plated MDA-MB-231 promotes the transition to an elongated shape with the extension of long protrusions. Interestingly both siRNA downregulation of DGKα and R59949-mediated inhibition impairs this change in shape (Fig. S3A to C) indicating the crucial requirement of DGKα activity.

**Figure 6:**
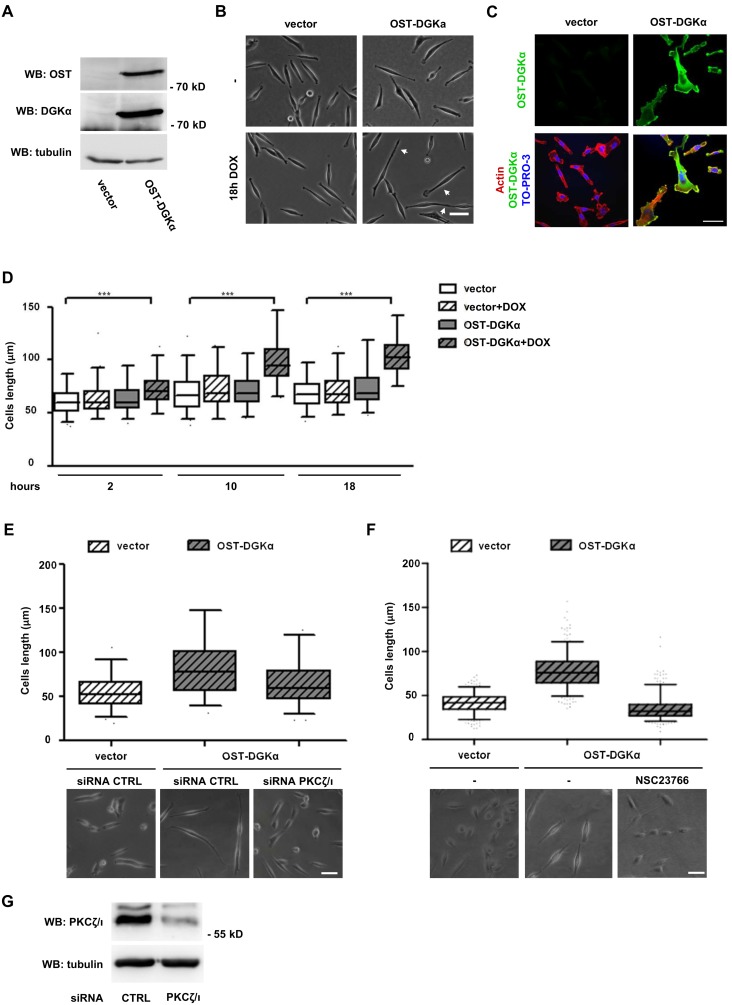
DGKα overexpression promotes a PKC-dependent cell elongation. MDA-MB-231 cells were infected with lentiviral vector expressing inducible OST-tagged DGKα or an empty vector. To induce DGKα expression, cells were treated overnight with doxycycline (1 µg/ml) in serum free medium. A) After cell lysis OST-DGKα induction was verified by western blotting with an antibody recognizing the OST-tag, while the extent of overexpression was verified with anti DGKα antibodies. Tubulin was used as loading control. B) Phase contrast images of control and OST-DGKα cells cultured in presence or absence of doxycycline. Arrows indicate cells with long protrusions. Scale bar 50 µm. C) Confocal images of doxycycline induced cells showing OST-DGKα localization, cells were stained for actin (red) and OST (green). Scale bar 24 µm. D) Time course of cell elongation at 2, 10 and 18 hours with or without doxycycline treatment. Time lapse videos were recorded and total cell length measured. Box and whiskers plots (black lines show median, whiskers: 5–95 percentile) of data from 3 independent experiments are shown, ***p<0.0001, 1 way ANOVA. E) MDA-MB-231 cells expressing OST-DGKα were transiently transfected with control or PKCζ/ι-specific siRNA. After 48 hours DGKα expression was induced by overnight treatment with doxycycline (1 µg/ml) in serum free medium. Images were acquired with a phase contrast microscope, representative images are shown. Scale bar 50 µm. Total cell length was measured for at least 100 cells and reported as box and whiskers plot. F) MDA-MB-231 cells expressing OST-DGKα were induced by overnight treatment with doxycycline (1 µg/ml) in serum free medium with or without NSC23766 (100 µM). Images were acquired with a phase contrast microscope, representative images are shown. Scale bar 50 µm. Total cell length was measured for at least 100 cells and reported as box and whiskers plot. MDA-MB-231 cells were transfected with CTRL and PKCζ/ι –specific siRNA and lysed. The efficiency of PKCζ/ι down–regulation by siRNA was verified by western blotting, tubulin was used as a loading control.

Since the over-expression of membrane-bound myr-DGKα stimulates cell migration in untransformed cells [18] and pseudopod extension and invasion in A2780 ovarian cancer cells [15], we investigated whether wild type DGKα over-expression was sufficient to further stimulate invasion in MDA-MB-231 cells. The previously described inducible OST-DGKα construct in MDA-MB-231 cells allowed us to verify this issue as doxycycline treatment induced a 30-fold increase in DGKα expression (Fig. 6A and Fig. S2A), with an increase of about 300-fold of the enzymatic activity (Fig. 2C). However, over-expression of OST-DGKα was not sufficient to enhance migration of MDA-MB-231 in wound-healing assay or to increase invasion through matrigel (Fig. S2B and C). Nevertheless, over-expression of OST-DGKα led to elongation of serum-starved MDA-MB-231 cells, while doxycycline did not affect the cell length of empty vector-infected MDA-MB-231 cells (Fig. 6B and D). Both in elongated and in shorter cells, OST-DGKα is localized at the tip of cell protrusions (Fig. 6C) suggesting that despite the absence of cytokines and growth factors the strong up-regulation of DGKα activity is sufficient to recruit the signaling machinery for membrane extension and to establish a feed forward loop recruiting further DGKα.

Consistently, with the reported role of the aPKCs in mediating DGKα-dependent Rac activation and membrane protrusions [23], we observed that siRNA-mediated silencing of aPKCs (Fig. 6G) blunted cell elongation induced by OST-DGKα over-expression (Fig. 6E). Also the Rac inhibitor NSC23766 completely blunted OST-DGKα induced elongation indicating the involvement of Rac family GTPases (Fig. 6F). Those findings confirm the relevance of aPKCs and Rac as DGKα downstream effectors promoting cytoskeletal remodeling and extension of membrane protrusions.

The expression of myr-DGKα drives pseudopodial extension by stimulating RCP-mediated recycling of β1 integrin in A2780 carcinoma cells [15]. However, siRNA-mediated silencing of either β1 integrin or RCP (Fig. S5C and D) did not affect protrusion elongation induced by wild type DGKα in serum starved MDA-MB-231 cells (Fig. S5A and B), suggesting that in this experimental model β1 integrin and its RCP-mediated recycling are not required for protrusion elongation.

These data indicate that up-regulation of DGKα activity by SDF-1α is sufficient to promote the extension of membrane protrusions through the aPKCs – RhoGDI – Rac pathway [22], [23], but that additional signaling pathways and/or its localization at specific myrstyoilation-directed membrane compartment are required to trigger cells invasion.

## Discussion

We and others established the relevance of DGKα activation and membrane recruitment in growth factors signaling [37]. In normal epithelia, endothelia and lymphocytes DGKα activity is required to convey proliferative [17], [38], [39] and migratory [16]–[18], [22], [23] signaling. Several studies pointed out DGKα involvement in cancer showing that its activity is necessary *in vivo* for glioblastoma and hepatocellular carcinoma progression [13], and *in vitro* for proliferation and survival of endometrial carcinoma [21], anaplastic large cell lymphoma [19], and melanoma [40]. Moreover, DGKα activity mediates matrix invasion sustained by p53 pro-metastatic mutations in cancer cells [15]. However, the molecular pathways by which DGKα controls carcinoma formation and metastatization are poorly known.

Inhere we investigated the role of DGKα in invasive signaling of SDF-1α, one of the key signals driving metastasis [41], whose receptor, CXCR4, is strongly associated to tumor growth and spontaneous metastasis formation [1]. We used MDA-MB-231 cells, a highly invasive human breast cancer cell line, whose invasiveness and tumorigenicity are dependent on the expression of SDF-1α receptor, CXCR4 [42]–[44]. In these cells we had previously shown that DGKα is required for EGF- [15] and HGF-induced [27] migration in a tridimensional environment.

Interestingly, we show here that DGKα is also regulated by SDF-1α, which stimulates its enzymatic activity and promotes its recruitment at ruffling sites (Fig. 2). Moreover, we show that activation of DGKα provides a key lipid signal required for SDF-1α pro-invasive activity in MDA-MB-231 cells (Fig. 1).

We previously showed that the PA generated by HGF-induced activation of DGKα recruits to the plasma membrane and activates aPKCs in a complex with RhoGDI and Rac1, thus mediating the release of Rac1 from RhoGDI, and its localization and activation at ruffle sites [23]. The aPKCs subfamily comprises the ζ and ι isoforms, which are activated by PA [28] but insensitive to DG.

Several pieces of evidence show that aPKCs and in particular PKCι, play a key role in cancer cell invasion and tumor progression [45]. Interestingly, PKCι is essential for K-Ras-driven invasion in colon cancer by regulating Rac1 [46], while aPKCs mediates EGF-induced cell migration of MDA-MB-231 breast cancer cells [47]. Altogether these data further suggest that the DGKα/aPKCs signaling axis contributes to pro-invasive signaling.

Accordingly, the finding that SDF-1α induces aPKCs localization at protrusion sites through activation of DGKα, indicates that the DGKα/aPKCs signaling axis mediates chemokine-driven mammary carcinoma invasiveness (Fig. 3). DGKα-dependent recruitment of aPKCs at protrusion is an essential signaling event, since the silencing of either DGKα or aPKCs impairs downstream events such as accumulation of β1 integrin and MMP-9 at the plasma membrane (Fig. 4 and 5). The functional relevance of aPKCs as a DGKα effector is further proved by the observation that its silencing impairs DGKα-induced cell elongation (Fig. 6E) and that its inhibition blocks SDF-1α-induced matrix invasion (Fig. 3F).

The findings that aPKCs, RCP and β1 integrin are all required for the invasiveness of MDA-MB-231 (Fig. 3F, 4H and ref. [15]), and that upon SDF-1α stimulation β1 integrin is concentrated at protrusion tips in a DGKα and aPKCs-dependent manner, are consistent with our previous data showing that DGKα-generated PA, through binding to RCP, docks α5β1 recycling vesicles to the tips of invasive pseudopods. Altogether these findings suggest that activation of aPKCs may also contribute to integrin recycling induced by chemokines and growth factors, although there is no experimental evidence for it.

Several pieces of evidence in different cell types indicate that activation of aPKCs regulates MMPs production and secretion [48]. For instance, PKCζ activation mediates MMP-9 secretion induced by SDF-1α in hematopoietic progenitors [11]. MMPs are key players in the tumor microenvironment and play a major role in invasion of extracellular matrix [49]. While some MMPs are transmembrane proteins, most of them are soluble and bind to the extracellular cell surface by interaction with several membrane proteins, including β1 integrin and CD44v [50]–[54].

Our finding that both DGKα and aPKCs are required for SDF-1α-induced release of MMP9 in the cell medium and for its accumulation at protrusions, provides further strength to our thesis that DGKα/aPKCs axis is a major component of chemokine pro-invasive signaling. Interestingly, in SDF-1α-stimulated cells, MMP-9 localization at cell surface superimposes with that of β1 integrin, suggesting that their function at protrusion tips is coordinately regulated by activation of DGKα/aPKCs signaling.

Finally, the observation that DGKα over expression drives by itself elongation of cell protrusions by regulating aPKCs is consistent with active PKCζ promoting wide cytoskeletal remodeling and protrusions in untrasformed cells [23]. The molecular mechanisms by which aPKCs induces cell elongation downstream to DGKα is still partially known. In line with our previous demonstration that activation of the DGKα/aPKCs signaling module stimulates the RhoGDI driven localization of both Rac1 and Cdc42 at membrane ruffles, we observed that the Rac inhibitor NSC23766 blunts DGKα induced cell elongation (Fig. 6G) and that SDF-1α-induced localization of Cdc42 at protrusions of MDA-MB-231 cells is significantly reduced by DGKα inhibition (Fig. S3D and E). Conversely, protrusion extension occurs even in the absence of β1 integrin and RCP, suggesting that DGKα-dependent activation of aPKCs regulates cytoskeletal remodeling independently from β1 integrin recycling and function, which are required, however, to enable cell migration through a 3D matrix (Fig. 4H). While it is clear that DGKα/aPKCs activity on cell elongation is independent on β1 integrin recycling, these data cannot rule out that accumulation of β1 integrin and MMP-9 at protrusion tips depends on DGKα/aPKCs-induced regulation of Rac1 or Cdc42 and cytoskeletal contractility [31].

Altogether we showed that activation of the DGKα/aPKCs/β1 integrin pathway plays a key role in chemokine-driven matrix invasion in breast cancer cells. Those observations suggest that DGKα inhibition or silencing could be effective not only in reducing primary tumor growth *in vivo* [13], [14] but could potentially also reduce the metastatic potential of carcinoma cells.

## Supporting Information

Figure S1 **DGKα is necessary for SDF-1α-induced cell invasion.** MDA-MB-231 cells were infected with lentiviral vectors expressing a shRNA against DGKα (shRNA-DGKα2) or an empty vector. A) Cells were lysed and the efficiency of DGKα down–regulation by shRNA was verified by western blot, tubulin was used as a loading control. B) 50,000 cells were plated on matrigel invasion chamber and incubated for 24 hours in presence or in absence of SDF-1α (100 ng/ml). Histogram reports mean ± SE of fold over control values from 3 independent experiments with *t-test p<0.05, ***t-test p<0.0005.
(TIF)

Figure S2 **DGKα overexpression does not affect migration and invasion of MDA-MB-231 cells.** MDA-MB-231 cells were infected with lentiviral vector expressing inducible OST-tagged DGKα or an empty vector. To induce DGKα expression, cells were treated overnight with doxycycline (1 µg/ml) in serum free medium. A) After cell lysis, the extent of DGKα overexpression was verified with anti DGKα antibodies, long and short exposures are shown. Actin was used as loading control. B) Cells were grown to confluence in 12 well plates and subjected to a wound healing assay for 24 hours in serum free medium. HGF (50 ng/ml) was used as a positive control. The cells were stained and those migrating inside 2.3 mm of wound counted. Histogram reports mean ± SE of fold over control values from 3 independent experiments with *t-test p<0.05. C) 50,000 cells were plated on matrigel invasion chamber and incubated for 24 hours in serum free medium. Medium with 10% FCS was used as positive control. Histogram reports mean ± SE of fold over control values from 3 independent experiments with *t-test p<0.05.
(TIF)

Figure S3 **DGKα is required for SDF-1α-induced pseudopod elongation.** A) MDA-MB-231 cells were plated on matrigel-coated coverslips for 20 hours in FCS containing medium, transfected with CTRL or DGKα -specific siRNA and cultured for further 20 hours in serum free medium. Cells were then stimulated for 6 hours with 50 ng/ml SDF-1α, fixed and photographed at phase contrast. B) Histogram reports protrusions length in µm as mean ± SE values of 4 independent experiments with *t-test p<0.005. C) MDA-MB-231 cells were plated on matrigel-coated coverslips for 20 hours in FCS containing medium and cultured for further 20 hours in serum free medium. Cells were then stimulated for 6 hours with 50 ng/ml SDF-1α, in presence or in absence of 1 µM R59949, fixed and photographed at phase contrast. Histogram reports protrusions length in µm as mean ± SE of 3 independent experiments with *t-test p<0.005. D) MDA-MB-231 cells were plated on matrigel-coated coverslips for 20 hours in FCS containing medium and cultured for further 20 hours serum free medium. Cells were stimulated for 6 hours with 50 ng/ml SDF-1α, in presence or in absence of 1 µM R59949, fixed and stained for actin (red) and Cdc42 (green). Arrowhead indicates Cdc42 at protrusions. Scale bar 24 µm. E) Histogram reports the percentage of cells displaying Cdc42 at protrusions as mean ± SE of 3 independent experiments with *t-test p<0.05.
(TIF)

Figure S4 **SDF-1α is not affecting surface exposition of β1-integrin and MMP-9.** A) Surface expression of β1 integrin was analyzed before (turquoise) and after (red) SDF-1α stimulation. Flow cytometry histogram overlay comparing the level of β1 integrin expression before and after SDF-1α expression. Isotype-matched controls mAb staining are given as dashed lines. MFI, median fluorescence intensity. B) Surface expression of MMP-9 was analyzed before (turquoise) and after (red) SDF-1α stimulation. Flow cytometry histogram overlay comparing the level of MMP-9 expression before and after SDF-1α expression. Isotype-matched controls mAb staining are given as dashed lines. MFI, median fluorescence intensity. C) MDA-MB-231 cells were plated on 6 wells dish for 20 hours in FCS containing medium and cultured for further 20 hours serum free medium. Cells were stimulated for 24 hours with 100 ng/ml SDF-1α, in presence or in absence of 1 µM R59949. MMP-9 mRNA was quantified by quantitative RT-PCR. Histogram reports the mean ± SE of 3 independent experiments.
(TIF)

Figure S5 **DGKα promoted cell elongation is independent from β1 integrin and RCP.** MDA-MB-231 cells were infected with lentiviral vector expressing inducible OST-tagged DGKα or an empty vector. A) Cells were transiently transfected with control or β1 integrin-specific siRNA. After 48 hours DGKα expression was induced by overnight treatment with doxycycline (1 µg/ml) in serum free medium. Images were acquired with a phase contrast microscope, representative images are shown. Scale bar 50 µm. Total cell length was measured for at least 100 cells and reported as box and whiskers plot. B) Cells were transiently transfected with control or RCP-specific siRNA. After 48 hours DGKα expression was induced by overnight treatment with doxycycline (1 µg/ml) in serum free medium. Images were acquired with a phase contrast microscope, representative images are shown. Scale bar 50 µm. Total cell length was measured for at least 100 cells and reported as box and whiskers plot. C) MDA-MB-231 cells were transfected with CTRL and β1 integrin-specific siRNA and lysed. The efficiency of β1 integrin down–regulation by siRNA was verified by western blotting, tubulin was used as a loading control. D) MDA-MB-231 cells were transfected with CTRL and RCP-specific siRNA and lysed. The efficiency of RCP down–regulation by siRNA and of OST-DGKα induction was verified by western blotting, actin was used as a loading control.
(TIF)
