# Supplementary figures and images for: The Diacylglycerol Kinase α/Atypical PKC/β1 Integrin Pathway in SDF-1α Mammary Carcinoma Invasiveness
Source: PLoS One. 2014 Jun 2;9(6):e97144. doi: 10.1371/journal.pone.0097144 (PMC4041662; doi:10.1371/journal.pone.0097144)

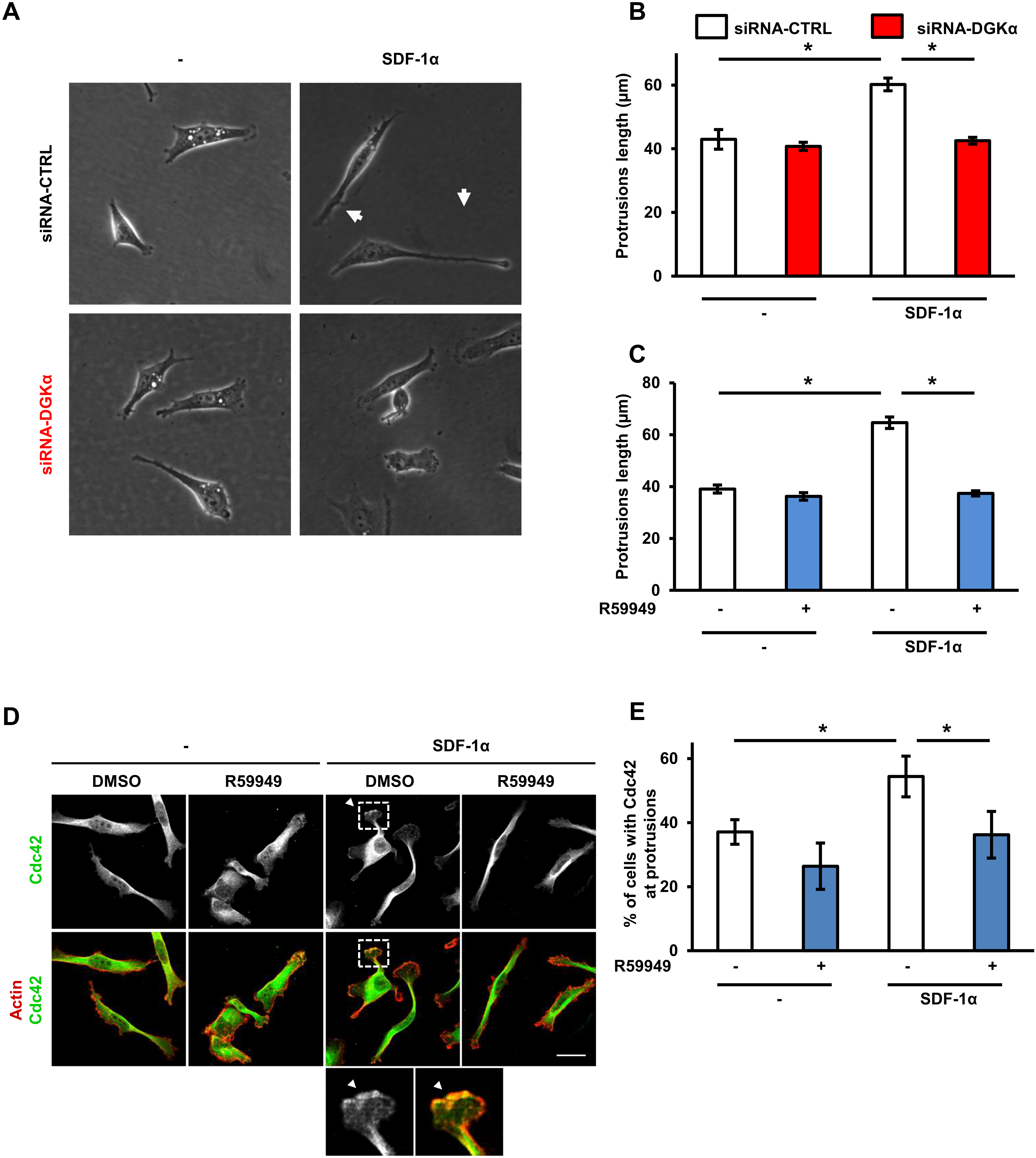

Supplement: Figure S3 — DGKα is required for SDF-1α-induced pseudopod elongation. A) MDA-MB-231 cells were plated on matrigel-coated coverslips for 20 hours in FCS containing medium, transfected with CTRL or DGKα -specific siRNA and cultured for further 20 hours in serum free medium. Cells were then stimulated for 6 hours with 50 ng/ml SDF-1α, fixed and photographed at phase contrast. B) Histogram reports protrusions length in µm as mean ± SE values of 4 independent experiments with *t-test p<0.005. C) MDA-MB-231 cells were plated on matrigel-coated coverslips for 20 hours in FCS containing medium and cultured for further 20 hours in serum free medium. Cells were then stimulated for 6 hours with 50 ng/ml SDF-1α, in presence or in absence of 1 µM R59949, fixed and photographed at phase contrast. Histogram reports protrusions length in µm as mean ± SE of 3 independent experiments with *t-test p<0.005. D) MDA-MB-231 cells were plated on matrigel-coated coverslips for 20 hours in FCS containing medium and cultured for further 20 hours serum free medium. Cells were stimulated for 6 hours with 50 ng/ml SDF-1α, in presence or in absence of 1 µM R59949, fixed and stained for actin (red) and Cdc42 (green). Arrowhead indicates Cdc42 at protrusions. Scale bar 24 µm. E) Histogram reports the percentage of cells displaying Cdc42 at protrusions as mean ± SE of 3 independent experiments with *t-test p<0.05. (TIF) [file pone.0097144.s003.tif]

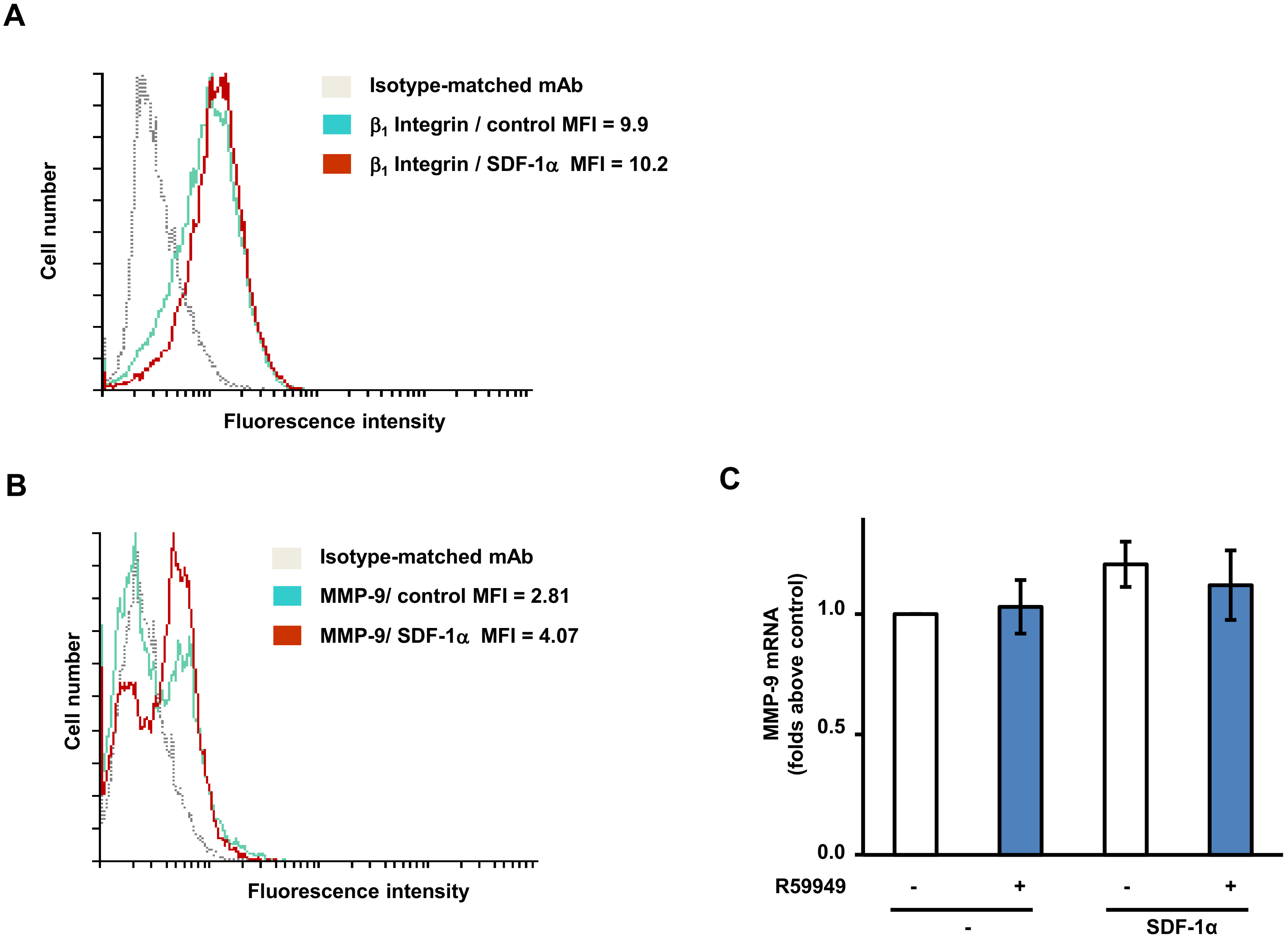

Supplement: Figure S4 — SDF-1α is not affecting surface exposition of β1-integrin and MMP-9. A) Surface expression of β1 integrin was analyzed before (turquoise) and after (red) SDF-1α stimulation. Flow cytometry histogram overlay comparing the level of β1 integrin expression before and after SDF-1α expression. Isotype-matched controls mAb staining are given as dashed lines. MFI, median fluorescence intensity. B) Surface expression of MMP-9 was analyzed before (turquoise) and after (red) SDF-1α stimulation. Flow cytometry histogram overlay comparing the level of MMP-9 expression before and after SDF-1α expression. Isotype-matched controls mAb staining are given as dashed lines. MFI, median fluorescence intensity. C) MDA-MB-231 cells were plated on 6 wells dish for 20 hours in FCS containing medium and cultured for further 20 hours serum free medium. Cells were stimulated for 24 hours with 100 ng/ml SDF-1α, in presence or in absence of 1 µM R59949. MMP-9 mRNA was quantified by quantitative RT-PCR. Histogram reports the mean ± SE of 3 independent experiments. (TIF) [file pone.0097144.s004.tif]
